# Supplementary material for: Control of Spreading Depression with Electrical Fields
Source: Sci Rep. 2018 Jun 8;8:8769. doi: 10.1038/s41598-018-26986-1 (PMC5993812; doi:10.1038/s41598-018-26986-1)
Supplement: Supplementary file 1 — Supplementary Information [file 41598_2018_26986_MOESM1_ESM.docx]

Title

Control of Spreading Depression with Electrical Fields

**Authors**

Andrew J. Whalen,^1,2^* Ying Xiao,^2,7^ Herve Kadji,^2,3^ Markus Dahlem,^8^ Bruce J. Gluckman,^2,3,4,5^ Steven J. Schiff^2,3,4,6^

**Affiliations**

^1^Mechanical Engineering, Pennsylvania State University, University Park, PA 16802.

^2^Center for Neural Engineering, Pennsylvania State University, University Park, PA 16802.

^3^Engineering Science and Mechanics, and ^4^Neurosurgery, Pennsylvania State University, University Park, PA 16802.

^5^Biomedical Engineering, Pennsylvania State University, University Park, PA 16802.

^6^Physics, Pennsylvania State University, University Park, PA 16802.

^7^Jiangsu Key Laboratory of Translational Research and Therapy for Neuro-Psycho-Diseases, Institute of Neuroscience, Department of Neurobiology, Soochow University, Suzhou, P. R. China.

^8^Physics, Humboldt University of Berlin, Berlin, Germany.

*Corresponding author: awhalen@psu.edu

**Supplementary Figures**


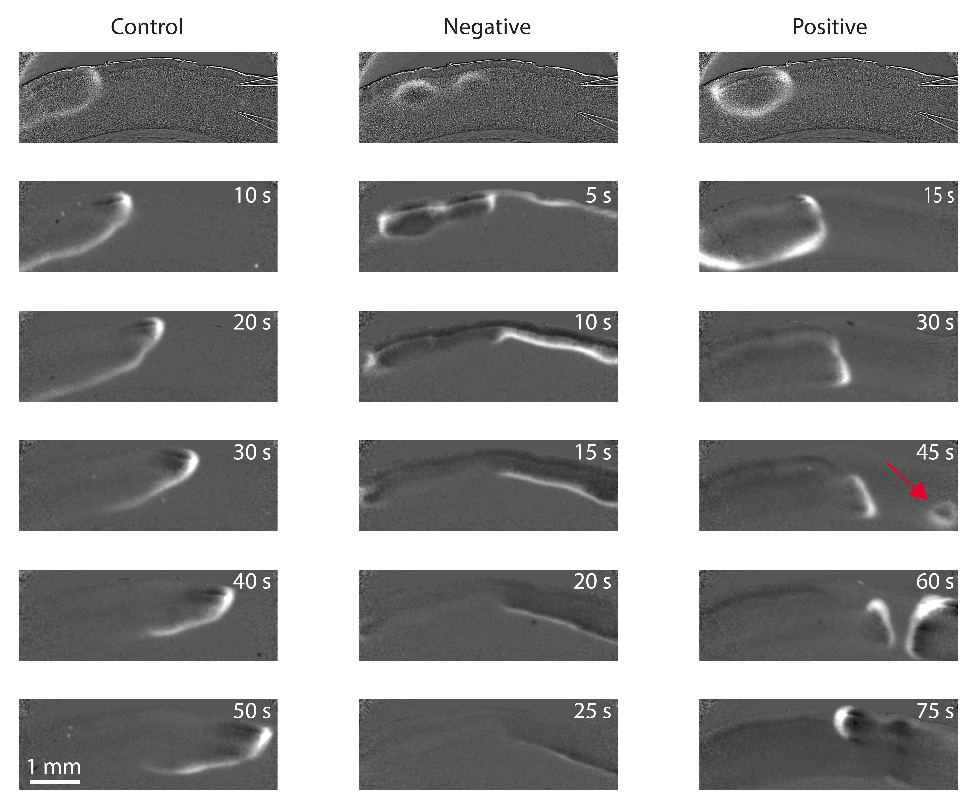


**Fig. S1. Electric field effects on the propagation and invasion of SD into the various layers of coronal slices evoked spontaneously in high K+ bath perfusion (26 mM), imaged via IOS.** Related to Figure 2. Normal SD propagation through all cortical layers during a control trial (left). SD propagation under surface-negative DC field (center) applied just before the second frame causes SD ignition to rapidly occur simultaneously across the uppermost cortical layers and propagation continues unabated. SD propagation was temporarily blocked from invading upper layers of cortex by an applied positive DC field (right), which in high K+ bath caused a secondary SD wave ignition in the deeper layers of cortex (red arrow). Eventually the continuous cellular depolarization from high K+ bath causes SD invasion to overcome the blocking effects of the positive field. Images contrast enhanced for display by background intensity subtraction.


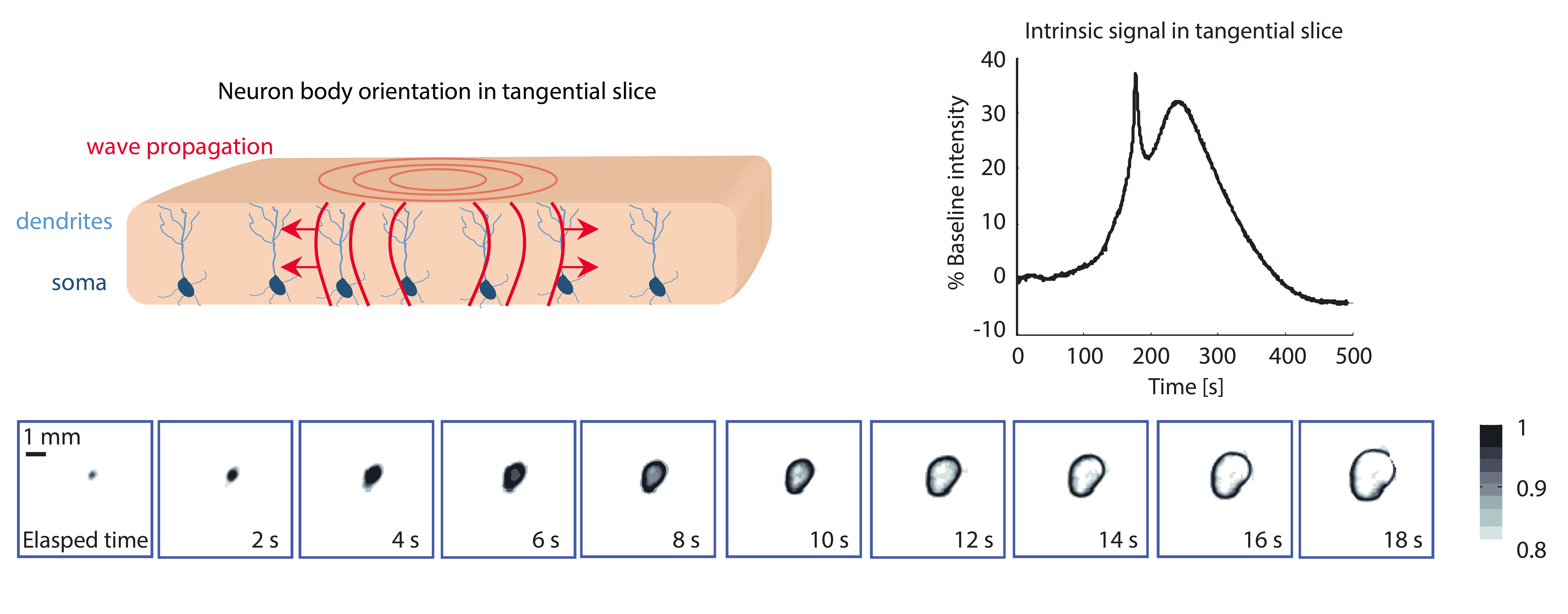


b

c

a

**Fig. S2. Summary of SD propagation in tangential slices.** Related to Figure S1. (**a**) Diagram of a tangential cortical slice and neuron body orientation demonstrating the typical propagation of SD and the near isotropic nature of tangential slices. (**b**) Intrinsic optical signal (IOS) recorded in tangential slices comprising a fast SD propagation related intensity peak followed by a slower recovery signal component. (**c**) IOS signal of ring wave propagation in tangential slices, darker color represents increasing depolarization.


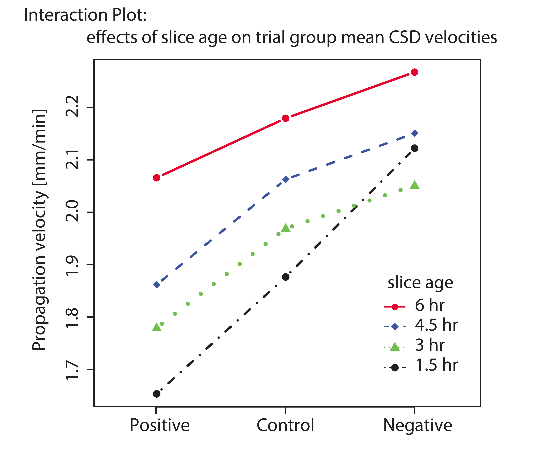


a

b

**Fig. S3. Statistical factors and data summary of SD propagation velocity.** Related to Figure 3. (**a**) Slice aging effects on the average propagation velocity of SD where each point represents an average of 13 slices. Note the consistency of slice age and polarity data factors as the overall trend between polarity groups remains intact as slice age varies. Slice aging results in faster SD propagation. (**b**) A normal quantile-quantile plot testing the normality of the propagation velocity data.

| **Table S1. Factorial ANOVA on Mean CSD Propagation Velocity.** Related to Figure 3. | | | | | | |
| --- | --- | --- | --- | --- | --- | --- |
|  | *Df* | *Sum Sq* | *Mean Sq* | *F value* | *P Value* | *Sig.* |
| Slice age | 3 | 3.839 | 1.2797 | 13.503 | 2.87e-08 | *** |
| Polarity | 5 | 3.545 | 0.7091 | 7.483 | 1.28e-06 | *** |
| Slice age\|Polarity | 15 | 0.742 | 0.0495 | 0.522 | 0.928 |  |
| Residuals | 288 | 27.293 | 0.0948 |  |  |  |
| Significance: p<0.001*** | | | | | | |

**Supplementary Videos**

**Video 1. Demonstration of surface-positive electric field arrest of spreading depression (bottom) compared to control (top).** Related to Figure 2. Video playback sped up 3x.

**Video 2. Demonstration of surface-negative electric field confinement of spreading depression to the superficial cortical layers (bottom) compared to control (top).** Related to Figure 2. Video playback sped up 3x.
